# Editorial: Transmission, colonization, and molecular pathogenesis of pneumococcus

**DOI:** 10.3389/fcimb.2022.1028047

**Published:** 2022-09-13

**Authors:** Jorge E. Vidal, Elsa N. Bou Ghanem, Xueqing Wu, Kaifeng Wu, Guangchun Bai, Sven Hammerschmidt

**Affiliations:** ^1^ Center for Immunology and Microbial Research, Department of Cell and Molecular Biology, University of Mississippi Medical Center, Jackson, MS, United States; ^2^ Department of Microbiology and Immunology, School of Medicine, University at Buffalo, Buffalo, NY, United States; ^3^ Department of Infectious Diseases, Sir Run Run Shaw Hospital, Zhejiang University School of Medicine, Hangzhou, China; ^4^ Department of Laboratory Medicine, The First People’s Hospital of Zunyi (The Third Affiliated Hospital of Zunyi Medical University), Zunyi, China; ^5^ Department of Immunology and Microbial Disease, Albany Medical College, Albany, NY, United States; ^6^ Department of Molecular Genetics and Infection Biology, Interfaculty Institute for Genetics and Functional Genomics, Center for Functional Genomics of Microbes, University of Greifswald, Greifswald, Germany

**Keywords:** *Streptococcus pneumoniae*, transmission, colonization, pathogenesis, invasive pneumococcal disease

*Streptococcus pneumoniae* (Spn; pneumococcus) has been for decades a number one bacterial killer of children and older adults worldwide, but it is also a commensal of the upper respiratory tract. Although vaccination with pneumococcal conjugate vaccines has decreased the burden of invasive pneumococcal disease (IPD), mortality caused by this pathogen remains a concern worldwide. The introduction of new generations of pneumococcal vaccines is creating a niche for vaccine-escape serotypes and changes in the microbiome of the upper airways is expected to occur. Moreover, the rise of multidrug-resistant clones around the world has posed a serious threat in recent years. A comprehensive understanding of the transmission, colonization, and molecular pathogenesis of the pneumococcus is necessary to come out with improved interventions aimed to further reduce the burden of IPD. To date, more than 100 distinct pneumococcal capsular serotypes have been identified but current pneumococcal conjugate vaccines (PCV10, PCV13, PCV20), and pneumococcal polysaccharide vaccine (PPSV23) protect against up to a total of 24 different pneumococcal types. These vaccines have decreased the burden of pneumococcal disease produced by vaccine types (VT) but provide poor protection against non-vaccine serotypes (NVT) and non-encapsulated Spn (NES) strains. Additionally, the increasing prevalence of NVTs, NES and multi-drug resistant Spn strains results in more challenge for the treatment of pneumococcal infections. In this Research Topic, we have compiled a series of research articles contributing to our understanding of transmission, colonization, molecular pathogenesis, the development of protein-based vaccines and antibiotic resistance; highlights from each study are described by the editors below.

An update on transmission in the current COVID-19 era was contributed by Willen et al.. Preventing strategies of the current COVID-19 pandemic, such as social distancing and wearing face masks, caused a reduced burden of IPD in Belgian children and the hypothesis was that transmission of pneumococci was interrupted. To gain insights into this, authors conducted a carriage surveillance in Belgium during the COVID-19 pandemic to find out that the overall pneumococcal carriage rate remained similar, comparing the data against a pre-COVID-19 period of the same children population.

Henares et al. studied the nasopharyngeal microbiota of children diagnosed with IPD and that were exposed to β-lactam antibiotics, compared to a cohort of children with IPD prior to antibiotic treatment. The authors found that antibiotic treatment increases oral bacteria and nosocomial bacterial species such as *Staphylococcus*, *Acinetobacter* and *Pseudomonas* with a transient decrease of streptococci phylogenetically related to Spn. Along the same lines, Spn forms a biofilm in the oropharynx and nasopharynx where it can interact with strains of the same species and other species. A study by Valente et al. thoroughly evaluated the interaction of phenotypically and genotypically unrelated Spn strains. As a result of intra-species interaction, their study identified four different outcomes: commensalism, competition, amensalism, and neutralism. Identifying ecological interactions in the upper airways and the molecular mechanisms of biofilm formation may guide us to future intervention studies aiming to reduce colonization by the pneumococcus.

Using pneumococcus as a model organism have provided remarkable insights to the field of genetics, but the mechanism by which Spn takes up naked and double-strand DNA is still under active investigation. In a study by Oliveira et al., authors demonstrated that the assemblage of the major pilin ComGC, the DNA receptor, is stabilized by ComGG and further identified a Glu5 residue (E5) as an essential residue to incorporate minor pilin proteins ComGD and ComGF to the major pilin subunit ComGC. Shedding light on our understanding of pneumococcal prophages, Martin-Galiano and Garcia conducted a genetic survey in >4,000 Spn genomes to identify full prophages in 43% of strains. The evolutionary relationship and putative role in pathogenesis were discussed by the authors and provide a valuable dataset to further study pneumococcal prophages.

Three review manuscripts highlight the advances on our understanding about conserved immunogenic proteins as vaccine targets. Aceil and Avci provide a thoughtful overview of multiple classes of conserved surface proteins utilized by pneumococcal strains as colonization or virulence factors. They approach the contribution of such surface proteins to colonization and IPD, and discuss data about the immune response of the human host. Then a paper by Gingerich and Mousa reviews the most recent information about the molecular mechanism of protection of those anti-pneumococcal antibodies. The authors discuss a variety of mechanisms including opsonophagocytic activity, toxin neutralization, and inhibition of bacterial adherence. Lane et al. focus their review paper on a family of proteins collectively known as choline-binding proteins (CBPs) and in particular on PspA, one of the most abundant CBP. They summarize new developments on pathogenesis and the potential use of CBPs and PspA for the treatment and prevention of pneumococcal pneumonia.

Exciting new molecular pathogenesis insights were published in a series of manuscripts. Takemura et al. identified a novel role for the enzyme β-galactosidase (BgaA) during pneumococcal bacteremia. Authors provide experimental evidence that BgaA indirectly induces tissue damage and triggers blood clot during sepsis. A paper by Mo et al. nicely provides an automated image analysis tool to evaluate the intercellular tight junction from *in vitro* and *in vivo* experiments. This new quantitative method is such a valuable tool to investigate invasion of epithelium and endothelium, through the disruption of tight junction by Spn strains but can be utilized with other pathogens as well. Along the same lines, Sura et al. used a co-infection model with Spn and H1N1 influenza virus to investigate modifications of the proteome or ubiquitome. However, their findings point out towards minor differences in ubiquitination abundance during Spn-influenza virus co-infection.

Selection of Spn during disease evolves rapidly and help Spn to successfully adapt to the new niche. Agnew et al. isolated two clones of the same strain from blood and cerebrospinal fluid of a child with meningitis. Molecular studies identified an altered raffinose utilization between the two strains that may influence Spn clones to cause meningitis. Using transcriptome and cytokine analysis, Moscardini et al. elegantly demonstrated a “recall” adaptive and innate immune response post-Spn infection. They identified an immune response signature including genes associated to the cytokine response. Finally, Ali et al. summarized the recent advances on the contribution of extracellular serine protease to pneumococcal disease and pathogenesis. The authors focus this review on the molecular and functional analysis of such pneumococcal enzymes and update the field with recent developments regarding their immunogenicity and interaction with the human host.

Despite new generations of pneumococcal vaccines and the development and FDA-approval of new antibiotics for IPD, the isolation of pneumococcal strains with resistance to first line antibiotics is on the rise. A study set up in a high-income country, Sweden, investigated the genetic relationship between their multidrug resistant (MDR) Spn strains and MDR global Spn isolates. Investigators identified global pneumococcal sequence cluster (GPSC) 1, GPSC9 and GPSC10, with the majority of their isolates belonging to one of these GPSCs. MDR, non-vaccine strains, were identified in GPSC9 and GPSC10 and warrants further surveillance as the burden of IPD caused by those vaccine escape strains may increase with the introduction of new generation of vaccines. Finally, Gonzales et al. investigated the burden of resistance in Peru, a low-middle income country post introduction of pneumococcal vaccines. Their stunning data revealed an increase macrolide resistance in both carriage strains and strains isolated from IPD cases in the country. Authors worry that a further increase in macrolide resistance can be observed in the years to come given that azithromycin is being empirically prescribed for COVID-19 cases worldwide.

The prevention and control of *S. pneumoniae* colonization and IPD are essential to global health. The sixteen articles published in this Research Topic have contributed to this pathogen’s research from different angles e.g., pneumococcal transmission, antibiotic resistance, genomics, molecular pathology, drug innovation, and reviewing recent findings on pneumcococal colonization and proteinaceous vaccine candidates. We believe these cutting-edge research outcomes expand our knowledge on Spn transmission and molecular mechanisms of colonization and will therefore significantly contribute to pneumococcal disease prevention and further new findings in this field.

## Author contributions

All authors contributed with the writing, and editing of the manuscript and approved the final version.

## Acknowledgment

JEV was in part supported by grants from the National Institutes of Health (NIH; 5R21AI144571-03 and 1R21AI151571-01). The content is solely the responsibility of the authors and does not necessarily represent the official view of the NIH. The work of SH is funded by grants from the Deutsche Forschungsgemeinschaft (DFG, German Research Foundation): DFG-RTG 2719 and DFG HA 3125/5-2 , the Bundesministerium für Bildung und Forschung (BMBF- Zwanzig20 -InfectControl 2020 – Project VacoME (FKZ 03ZZ0816A), Pathowiki (FKZ 03ZZ0839B) and Pneumofluidics (FKZ 01DP19007).

## Conflict of interest

The authors declare that the research was conducted in the absence of any commercial or financial relationships that could be construed as a potential conflict of interest.

## Publisher’s note

All claims expressed in this article are solely those of the authors and do not necessarily represent those of their affiliated organizations, or those of the publisher, the editors and the reviewers. Any product that may be evaluated in this article, or claim that may be made by its manufacturer, is not guaranteed or endorsed by the publisher.

